# Fed-batch SSCF using steam-exploded wheat straw at high dry matter consistencies and a xylose-fermenting *Saccharomyces cerevisiae* strain: effect of laccase supplementation

**DOI:** 10.1186/1754-6834-6-160

**Published:** 2013-11-13

**Authors:** Antonio D Moreno, Elia Tomás-Pejó, David Ibarra, Mercedes Ballesteros, Lisbeth Olsson

**Affiliations:** 1 Instituto IMDEA Energía, Biotechnological Processes for Energy Production Unit, Móstoles 28935, Spain; 2 Department of Chemical and Biological Engineering, Industrial Biotechnology, Chalmers University of Technology, Göteborg SE-41296, Sweden; 3 INIA-CIFOR, Forestry Products Department, Cellulose and Paper Laboratories, Madrid 28040, Spain; 4 Centro de Investigaciones Energéticas, Medioambientales y Tecnológicas, Biofuels Unit, Madrid 28040, Spain

**Keywords:** Lignocellulose, Bioethanol, Simultaneous saccharification and co-fermentation, *In situ* laccase detoxification, Steam explosion, Xylose-fermenting *Saccharomyces cerevisiae*

## Abstract

**Background:**

Lignocellulosic bioethanol is expected to play an important role in fossil fuel replacement in the short term. Process integration, improvements in water economy, and increased ethanol titers are key considerations for cost-effective large-scale production. The use of whole steam-pretreated slurries under high dry matter (DM) conditions and conversion of all fermentable sugars offer promising alternatives to achieve these goals.

**Results:**

Wheat straw slurry obtained from steam explosion showed high concentrations of degradation compounds, hindering the fermentation performance of the evolved xylose-recombinant *Saccharomyces cerevisiae* KE6-12 strain. Fermentability tests using the liquid fraction showed a higher number of colony-forming units (CFU) and higher xylose consumption rates when treating the medium with laccase. During batch simultaneous saccharification and co-fermentation (SSCF) processes, cell growth was totally inhibited at 12% DM (w/v) in untreated slurries. However, under these conditions laccase treatment prior to addition of yeast reduced the total phenolic content of the slurry and enabled the fermentation. During this process, an ethanol concentration of 19 g/L was obtained, corresponding to an ethanol yield of 39% of the theoretical yield. By changing the operation from batch mode to fed-batch mode, the concentration of inhibitors at the start of the process was reduced and 8 g/L of ethanol were obtained in untreated slurries with a final consistency of 16% DM (w/v). When fed-batch SSCF medium was supplemented with laccase 33 hours after yeast inoculation, no effect on ethanol yield or cell viability was found compared to untreated fermentations. However, if the laccase supplementation (21 hours after yeast inoculation) took place before the first addition of substrate (at 25 hours), improved cell viability and an increased ethanol titer of up to 32 g/L (51% of the theoretical) were found.

**Conclusions:**

Laccase treatment in SSCF processes reduces the inhibitory effect that degradation compounds have on the fermenting microorganism. Furthermore, in combination with fed-batch operational mode, laccase supplementation allows the fermentation of wheat straw slurry at high DM consistencies, improving final ethanol concentrations and yields.

## Background

In terms of volume and market value, biomass is considered to be the most important source of renewable energy and bioethanol is considered to be the main alternative for fossil fuel replacement in the transportation sector in the short to medium term [1].

Lignocellulosic biomass is an abundant, low-cost, and widely distributed feedstock that can be used as raw material for the production of biofuels and other value-added products in future biorefineries. Biochemical conversion of lignocellulose to ethanol is, however, hindered by the complex and recalcitrant structure of these materials. To increase biomass digestibility, several pretreatment methods have been developed, with hydrothermal technologies such as steam explosion being one of the most commonly used [2,3]. During steam explosion, the accessibility of enzymes to cellulose is increased due to the solubilization of hemicelluloses and the redistribution of lignin. Inevitably, different by-products (weak acids, furan derivatives, and phenolic compounds) that inhibit enzymes and fermentation microorganisms in the subsequent steps are also generated in the process [4-7]. The inhibitory levels partly depend on fermentation variables including the physiological conditions of the cell, the dissolved oxygen tension, and the pH of the medium. Thus, by adjusting the fermentation conditions the inhibitory effects can be alleviated.

There are different ways to overcome the effects of the inhibitory compounds. One of the most commonly used strategies to avoid inhibition is to remove the inhibitors from the slurry using physical, chemical, or biological detoxification methods. Of the different biological detoxification strategies, *in situ* laccase treatment has been considered a more feasible option than other methods because such treatment does not require extra equipment and it is performed under mild reaction conditions [8]. Laccase enzymes have been purified from different ligninolytic microorganisms, mainly white-rot fungi, and they can oxidize substituted phenols, anilines, and aromatic thiols by reducing oxygen to water [9]. A wide range of pretreated materials have been subjected to laccase detoxification, resulting in improved fermentability after the treatment [10-14].

Agricultural residues such as wheat straw have been shown to be promising feedstocks for future biorefineries. Wheat straw is a very suitable material for bioethanol production because it is composed of high amounts of glucose and xylose, the latter being of utmost importance when xylose-fermenting yeasts are available. *Saccharomyces cerevisiae* KE6-12 (modified with *XYL1* and *XYL2* genes encoding xylose reductase (XR) and xylitol dehydrogenase (XDH) from *Pichia stipitis*, respectively), which can consume both glucose and xylose from lignocellulosic materials in the presence of degradation compounds, represents a good choice as this strain has been shown to consume xylose completely with a 90% theoretical ethanol yield even on a demonstration scale (10 m^3^) [15].

There is no doubt about the need to increase the substrate loading in the fermentation process to reach higher ethanol concentrations and make the process economically viable. Even so, the use of whole slurries at high substrate loading will mean higher amounts of degradation products, in which case the use of strains that are highly inhibitor tolerant would be crucial. In the present study, the authors combined the use of the evolved xylose-recombinant *S. cerevisiae* KE6-12 strain with laccase enzyme treatment, with the purpose of increasing the final ethanol concentration using whole wheat straw slurry. Batch and fed-batch SSCF processes were run under high dry matter (DM) conditions to compare the ethanol concentrations, sugar consumption, and cell viability in untreated and laccase-treated fermentations.

## Results and discussion

### Steam-explosion pretreatment

Pretreated material was characterized and its composition is shown in Table 1. After steam explosion, the collected wheat straw slurry had a total DM content of 26% (w/v), 21.5% (w/v) of which was water-insoluble solids (WIS). The WIS fraction of the slurry was mainly composed of cellulose (47.4% w/w) and lignin (25.4% w/w), with minor hemicellulose content (8.4% w/w) (Table 1).

**Table 1:** Composition of pretreated wheat straw

**Compound**	**Pretreated material (210°C, 2.5 minutes)**^**a**^
*WIS (% (w/w))*	
Cellulose	47.4
Hemicellulose	8.4
Lignin	25.4
Others	1.5
*Liquid fraction (% (w/w))*	
Glucan	1.0
Glucose	0.3
Xylan	6.6
Xylose	0.7
Arabinan	0.1
Arabinose	0.4
Galactan	0.4
Galactose	0.2
Total phenol	2.6
Acetic acid	1.6
Formic acid	2.1
Furfural	0.19
5-HMF	0.07

^a^Values expressed as g/100 g of slurry (dry weight). 5-HMF, 5-hydroxymethylfurfural; WIS, water-insoluble solids.

As a consequence of hemicellulose solubilization, a high xylose concentration (mostly in the oligomeric form) was measured and different degradation products were identified and quantified as soluble compounds (Table 1). These compounds are considered to be inhibitors and can affect biochemical pathways in the fermenting microorganisms and interact with cellulolytic enzymes, leading to reduced final ethanol titers and volumetric productivities [6,7,16,17]. Acetic acid, formic acid, and phenolic compounds were the most abundant degradation products in terms of percentage (w/w) of total slurry (Table 1). Acetic acid is derived from the acetyl groups present in hemicelluloses [18]. At low pH in the fermentation medium, the acetic acid (pKa = 4.7) is in the undissociated form, is liposoluble, and diffuses into the cells. Inside the cell (pH = 7.4), the acid dissociates causing a decrease in pH that inhibits different activities and promotes an energy imbalance by removing these ions through ATP pumps [16]. Since the formation of acetic acid is inherent to hemicellulose hydrolysis, its formation cannot be prevented. Furfural and 5-hydroxymethylfurfural (5-HMF) were also identified in the slurry, and they are produced by pentose and hexose dehydration, respectively. These compounds affect cell growth and respiration rates, and most yeast used for ethanol production can reduce the aldehyde group on the furan ring to convert them into less toxic alcoholic forms [17]. The ability of yeasts to transform furfural and 5-HMF offers a way of *in situ* detoxification. To some extent, this encourages resistance to furans, or the yeasts may gradually become adapted to their presence. Further degradation of furfural and 5-HMF generates formic acid, which has similar inhibitory action to that of acetic acid. In addition to the inhibitors already mentioned, a variety of aromatic, polyaromatic, phenolic, and aldehydic compounds are released from the lignin fraction [18,19]. Among them, a wide variety of substituted phenols and cinnamic acids such as vanillin, syringaldehyde, 4-hydroxybenzaldehyde, ferulic acid, and *p*-coumaric acid have previously been reported to be present in steam-exploded wheat straw [19-21]. These compounds can cause partitioning and loss of integrity of cell membranes, reducing both the specific growth rate and the assimilation of sugars. Phenols, especially low-molecular-weight compounds, have a considerable inhibitory effect and are more toxic than furfural and 5-HMF (even at low concentrations), and their effects are difficult to alleviate by adjusting the fermentation conditions [17,18]. In addition to this, phenolic compounds can also inhibit and deactivate hydrolytic enzymes [6,7].

### Effect of laccase on the liquid fraction fermentation

**Figure 1:**
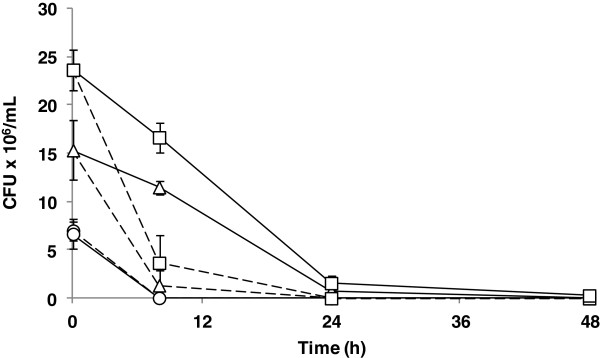
**Fermentation of diluted wheat straw liquid fraction equivalent to 14% DM (w/v).** Time course of cell viability in terms of CFU/mL during the fermentation process of untreated liquid fraction (discontinuous lines) and laccase-treated liquid fraction (continuous lines) at 14% DM (w/v) with inoculum sizes of 1 g/L (○), 3 g/L (△), and 5 g/L (□). CFU, colony-forming unit; DM, dry matter.

In order to evaluate the fermentability of the pretreated material and the ability of the evolved recombinant strain to ferment xylose in the presence of degradation compounds, the liquid fraction from pretreated slurry was diluted to concentrations corresponding to different DM content and fermented using different inoculum sizes (1, 3, and 5 g/L). Moreover, to test the effects of laccase on the fermentability of the liquid fraction, *Pycnoporus cinnabarinus* laccase was added to the culture medium at 1 IU/mL. The number of colony-forming units (CFU/mL) dropped rapidly in untreated liquid fractions equivalent to 14% DM content, independently of inoculum size, showing that the toxic compounds had a fatal effect on the fermenting microorganism (Figure 1). When laccase treatment was used, 73% of the phenol content was removed (the concentration decreased from 4.8 to 1.3 g/L) and an increase in the inoculum size from 1 to 3 or 5 g/L resulted in maintenance of cell viability after 24 hours from inoculation. The concentration of weak acids or furan derivatives was not affected by the laccase treatment (data not shown), which has also been seen in other investigations [10,14,21,22].

Detoxification by laccases implies an oxidative polymerization mechanism. Laccases extract one electron from phenols, generating unstable phenoxy radicals that can interact with each other, leading to polymerization into large-size aromatic compounds, which are less toxic for fermentative microorganisms [10]. During this process, the selective action of laccases on different phenols establishes a faster conversion for syringaldehyde or cinnamic acids, while vanillin is oxidized at lower rates and other compounds such as 4-hydroxybenzaldehyde are not affected [20,22]. Thus, non-laccase-active compounds or those that are oxidized at lower rates can remain in the medium and, together with weak acids and furan derivatives, have an inhibitory effect on fermentative strains, mainly reducing the volumetric ethanol productivity.

**Figure 2:**
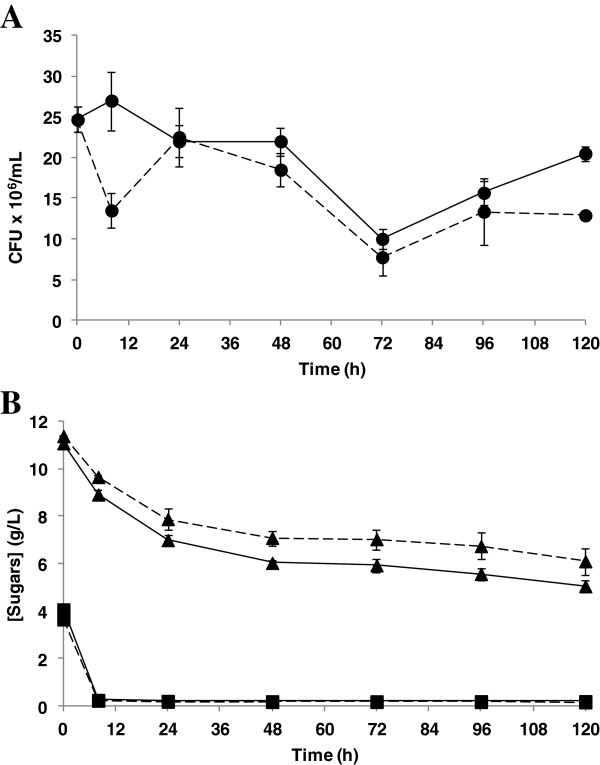
**Fermentation of diluted wheat straw liquid fraction equivalent to 12% DM (w/v).** Time course for the fermentation process of untreated liquid fraction (discontinuous lines) and laccase-treated liquid fraction (continuous lines). **(A)** Cell viability (●) in terms of CFU/mL. **(B)** Glucose (■) and xylose (**▲**) concentration. CFU, colony-forming unit; DM, dry matter.

To improve cell viability and xylose consumption, the liquid fraction was diluted to the equivalent of 12% DM content and inoculated with 5 g/L of evolved *S. cerevisiae* KE6-12, because of the best growth at this inoculum size when using the liquid fraction corresponding to 14% DM. Due to the lower concentration of inhibitors in the fermentation medium at the equivalent to 12% DM, the cell viability was maintained throughout the 120-hour long process, even when no laccase treatment was given (Figure 2A). Glucose was the first sugar to be depleted in both untreated and laccase-treated liquid fractions, and no differences in consumption rates were found (Figure 2B). Following glucose consumption, xylose was consumed at lower rates and a shorter lag phase for laccase-treated liquid fractions led to higher xylose consumption rates at the initial stage of fermentation. Nevertheless, analysis of variance (ANOVA) did not show statistically significant differences between the ethanol yields at the 95.0% confidence level. The reduction in the lag phase can be attributed to the 81% decrease in phenolic content by the action of laccase (Table 2). As explained above, phenolic compounds can alter biological membranes, thus affecting the growth of fermenting microorganisms [16]. Lower amounts of soluble phenols during fermentation favor cell growth and better ethanol yield [11-14].

**Table 2:** Initial phenols, glucose, and xylose, and maximum ethanol concentration and ethanol yield

**Liquid fraction (equivalent % (w/v))**		**Phenols**_**ini**_**(g/L)**	**EtOH**_**max**_**(g/L)**	**Glucose**_**ini**_**(g/L)**	**Xylose**_**ini**_**(g/L)**	**Y**_**E/S**_**(g/g)**^**a**^
12% DM	Untreated	4.2	3.7	3.7	11.4	0.25^b^
	Laccase	0.8	4.2	4.1	11.1	0.28^b^

^a^Ethanol yield based on total sugars measured prior to fermentation; ^b^no statistically significant differences. Fermentation of liquid fraction with an inoculum size of 5 g/L. DM, dry matter; EtOH, ethanol; ini, initial; max, maximum.

### SSCF of wheat straw slurry in batch mode

**Figure 3:**
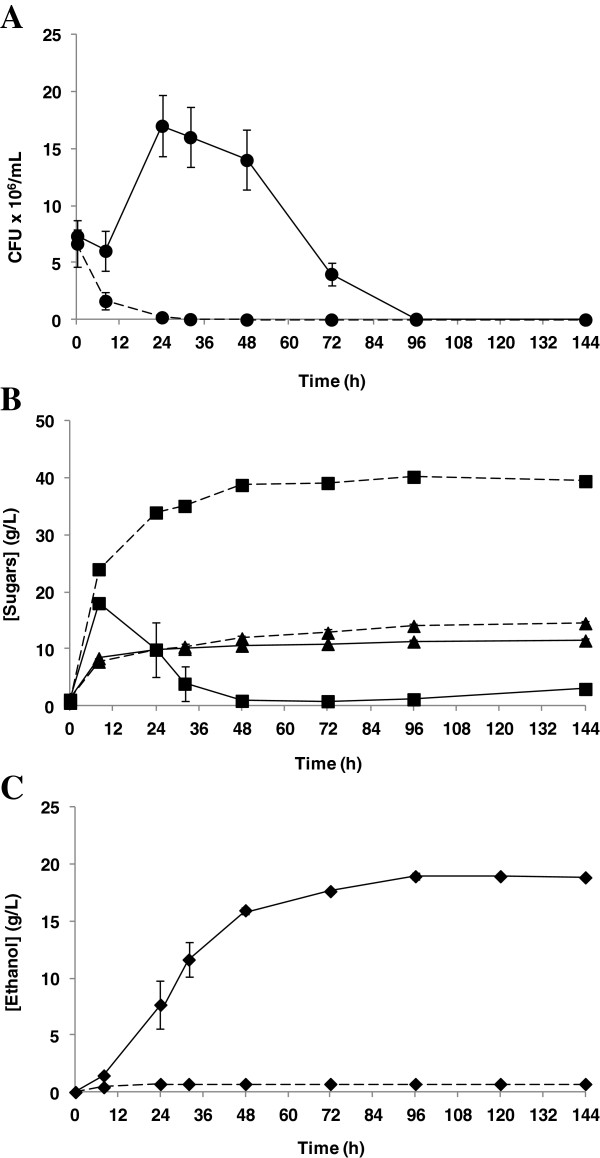
**Batch SSCF processes of wheat straw slurry at 12% DM (w/v).** Time course for batch SSCF process of untreated slurry (discontinuous lines) and laccase-treated (continuous lines) slurry. **(A)** Cell viability (●) in terms of CFU/mL. **(B)** Glucose (■) and xylose (**▲**) concentrations. **(C)** Ethanol (♦) concentration. CFU, colony-forming unit; DM, dry matter; SSCF, simultaneous saccharification and co-fermentation.

Taking into account the results obtained during the fermentation of the liquid fraction, 12% DM (w/v) diluted slurries were subjected to SSCF processes in batch mode. In this case, the inoculum size of *S. cerevisiae* KE6-12 was reduced to 1 g/L in order to minimize this parameter and enable detection of differences after laccase treatment. As illustrated in Figure 3A, cell viability was lost within the first 24 hours and neither sugar consumption nor ethanol concentration was observed in untreated slurries (Figure 3B,C). On the other hand, laccase treatment prior to the SSCF process reduced the amount of soluble phenols in slurries, allowing growth of the fermenting microorganism, and a maximum number of CFU/mL was reached at 24 hours. Laccase-treated slurries had 77% less phenols than untreated ones. This value was slightly lower than the observed one for the liquid fraction, even when the treatment was 9 hours longer. This result could be explained by mixing problems when using diluted slurry [23]. Limitation in terms of mixing and mass transfer can affect the homogeneity of the slurry and diminishing the accessibility of phenols to the action of laccase [20,24].

When the substrate consistency was increased from 12% to 16% DM (w/v), no cell growth was observed, even in laccase-treated slurries, due to the high concentration of inhibitory compounds and greater mass transfer limitations. The use of laccases for the detoxification of wheat straw slurry has already been described. Jurado *et al*. (2009) [12] found an increase in ethanol concentration of 2 to 2.7 times when enzymatic hydrolysates from both acid and non-acid steam-pretreated materials were detoxified with laccases from *Coriolopsis rigida* and *Trametes villosa*. In the same way, Moreno *et al*. (2012) [14] used laccases from *P. cinnabarinus* and *T. villosa* to detoxify the whole slurry from steam-exploded wheat straw, allowing the thermotolerant yeast *Kluyveromyces marxianus* CECT 10875 to ferment diluted slurries that could not be fermented before treatment. Furthermore, these authors have also reported an ethanol concentration of up to 22 g/L when laccase-treated slurries were fermented with the xylose-fermenting *S. cerevisiae* F12 strain, even in the absence of an external nitrogen source [24].

Due to the lag phase shown by *S. cerevisiae* KE6-12 after inoculation in batch SSCF at 12% DM (w/v), glucose and xylose concentrations increased at the start of the process (Figure 3B). The presence of both glucose and xylose usually results in a delay in xylose consumption during co-fermentation, as these sugars share the transporter by which they are transported into the cells [25]. Thus, a preference for glucose over xylose can limit the consumption of xylose, therefore explaining its higher concentration at the end of the fermentation. A final ethanol concentration of 19 g/L was obtained in laccase-treated slurries (Figure 3C). This value corresponds to a final ethanol yield of 0.20 g/g based on the total amount of glucose and xylose from the solid and liquid fractions of the diluted slurries (glucose from enzyme preparations was also taken into account). Assuming a theoretical ethanol yield of 0.51 g/g for both sugars, 39% of the maximum amount of possible ethanol resulted under these conditions. However, when only the sugars released during saccharification were considered (with values determined from untreated slurries), the final yield increased to 70%, showing the enzymatic hydrolysis to be an important limiting factor. An efficient saccharification step is essential to obtain higher ethanol concentrations. Development of new enzymatic cocktails with improved activities can contribute to better hydrolytic performances, increasing the availability of sugar during fermentation [26,27]. In addition, the use of thermotolerant strains that can ferment at temperatures close to the optimal for saccharification could improve this step, thus contributing to increase final ethanol concentrations [19,28].

### SSCF of wheat straw slurry in fed-batch mode

For cost-effective bioethanol production, ethanol concentrations above 4% (w/v) are needed to reduce distillation costs [29]. By increasing substrate loadings, higher concentrations of fermentable sugars are available and then higher ethanol concentrations can be achieved [30]. Increasing the solids content, however, would also lead to higher concentrations of inhibitory compounds, which would explain why batch SSCF at 16% DM (w/v) could not be fermented. One feasible alternative to achieve higher substrate loadings is to change the operational mode from batch to fed-batch. Under the latter configuration, the substrate is gradually added to the medium, keeping the concentration of inhibitors at levels suitable for fermentation, and *in situ* adaptation of microorganisms to inhibitory compounds could improve their tolerance towards a new substrate addition. Moreover, low initial substrate loadings avoid mixing problems and reduce accumulation of glucose, so a more efficient co-fermentation of xylose and glucose takes place [31,32].

**Figure 4:**
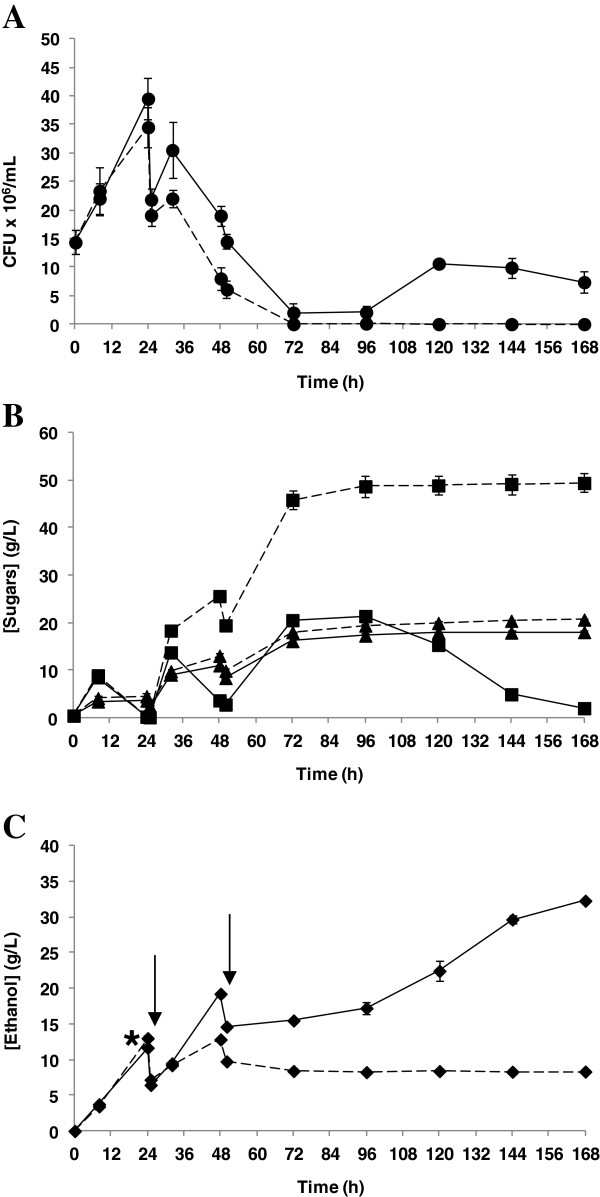
**Fed-batch SSCF processes of wheat straw slurry at 16% DM (w/v).** Time course for fed-batch SSCF process of untreated slurry (discontinuous lines) and laccase-treated slurry (continuous lines) (supplemented after 21 hours of inoculation). **(A)** Cell viability (●) in terms of CFU/mL. **(B)** Glucose (■) and xylose (**▲**) concentrations. **(C)** Ethanol (♦) concentration. The asterisk and arrows indicate addition of laccase and substrate, respectively. CFU, colony-forming unit; DM, dry matter; SSCF, simultaneous saccharification and co-fermentation.

The fed-batch assays were performed with an initial slurry content of 6% DM (w/v) and an inoculum size of 3 g/L (but when considering all substrate additions, the inoculum size was 1.2 g/L). After 25 hours and 50 hours of fermentation, slurry was added, reaching a substrate consistency of 12% and 16% DM (w/v), respectively. When using this fed-batch strategy, ethanol at 8.3 g/L was produced in untreated slurries (Figure 4C). It is remarkable that no fermentation took place in batch mode at the same consistency. As mentioned above, *in situ* adaptation of the fermenting microorganism may take place during the fed-batch assay (Figure 4A,B). While fermentation at 12% DM (w/v) in batch mode was not possible for untreated slurries, a continuous increase in the ethanol concentration was observed when the same substrate consistency (after the first addition of substrate) was reached with the fed-batch strategy. However, after the second addition of substrate, cell growth ceased completely and the production of ethanol (and sugar consumption) suddenly stopped, resulting in accumulation of sugars due to the continued enzymatic action.

During fed-batch SSCF assays, laccase was added using different strategies. In one set of experiments, laccase was added 4 hours before the first addition of substrate and in a second set of experiments, it was added 8 hours after the first addition (at 21 hours and 33 hours of fermentation, respectively). In contrast to batch SSCF, a laccase treatment step prior to inoculation in fed-batch SSCF was not performed since the amounts of inhibitory compounds were low at the start of the process. Furthermore, it has been shown that laccase treatment prior to enzymatic hydrolysis reduces the saccharification yield of steam-exploded wheat straw [12,14,24]. Thus, a delay in addition of laccase can reduce this negative effect on enzymatic hydrolysis and minimize the effect of laccase treatment on saccharification yields. When laccase was added 21 hours after inoculation, the phenolic content during fermentation remained below 0.6 g/L after detoxification (with total phenolic content measured at 32, 48, and 72 hours after yeast inoculation). As a result, cell viability was enhanced, reaching the maximum CFU number at 24 hours and keeping the cells alive throughout the fermentation (Figure 4A). In terms of product concentration, 32.3 g/L ethanol were found at the end of the process (Figure 4C), corresponding to an ethanol yield of 0.26 g/g (51% of theoretical). This yield was 1.3 times higher than that obtained with laccase-treated slurries at 12% DM (w/v) in batch mode, even when using higher substrate consistency (16% DM (w/v)). An increase of the ethanol concentrations and yields when working with steam-exploded wheat straw in a fed-batch operation mode SSCF was also observed by other authors [19,31]. Tomás-Pejó *et al*. (2009) [19] reported an ethanol concentration of 36.2 g/L and ethanol yield of 0.33 g/g with a fed-batch strategy (10% WIS (w/v) as initial substrate concentration that was increased until 14% WIS (w/v) after 12 hours), compared with an ethanol concentration and yield of 30.2 g/L and 0.27 g/g, respectively, in batch strategy at 14% WIS (w/v). In a similar way, Olofsson *et al*. (2008) [31] showed an increase from 59% in batch mode up to 71% in fed-batch mode, of the theoretical ethanol yield, using steam-pretreated wheat straw at a final WIS content of 9%.

The higher ethanol yields in fed-batch SSCF can be explained due to the better saccharification yields during the fed-batch processes as in this operational mode the initial substrate consistencies are low. By increasing substrate consistency, a decrease in saccharification yields has previously been described due to end-product inhibition, unproductive binding, protein deactivation or denaturalization, and the decline in the binding capacity of enzymes to cellulose [33-35]. In this context, a lower initial substrate concentration could increase enzymatic hydrolysis yields, resulting in higher overall yields. Furthermore, since the inhibitors are kept at a lower level than in batch SSCF, higher co-consumption of glucose and xylose can be expected, considering also the fact that low glucose concentration at early stages of the process facilitates xylose consumption [15,31].

Either in untreated or laccase-treated slurry, an adaptation of the microorganism to the higher concentrations of inhibitors after each addition of substrate was required. This adaptation phase was more remarkable at critical stages (12% DM (w/v) for untreated slurry and 16% DM (w/v) for laccase-treated slurry). During these adaptation phases, ethanol was produced at lower rates and glucose accumulated in the medium (Figure 4B,C).

**Figure 5:**
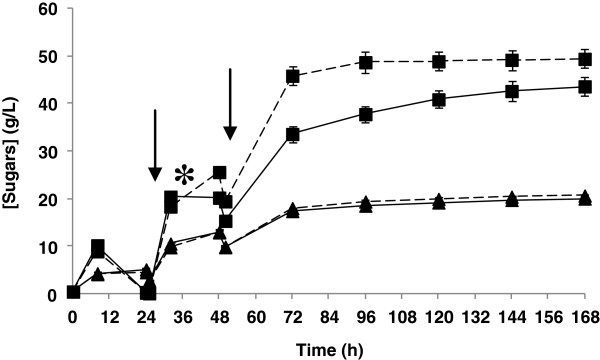
**Profile of sugars during fed-batch SSCF process of wheat straw slurry at 16% DM (w/v).** Saccharification profiles during a fed-batch SSCF process with untreated wheat straw slurry (discontinuous lines) and laccase-treated wheat straw slurry (continuous lines). Glucose (■) and xylose (**▲**) concentrations. The asterisk and arrows indicate addition of laccase and substrate, respectively. DM, dry matter; SSCF, simultaneous saccharification and co-fermentation.

In a second set of experiments, laccase was added after the first addition of substrate, at 33 hours after inoculation. Surprisingly, laccase supplementation resulted in the same ethanol concentration and cell viability profiles as those obtained with untreated slurries. Thus, after the second addition of substrate (50 hours), cell growth and ethanol concentration were not observed (data not shown), and glucose and xylose accumulated (Figure 5). Furthermore, a considerable difference in glucose concentration was observed between untreated and laccase-treated slurries. This result is in accordance with previous data indicating a clear effect in the saccharification step of steam-exploded wheat straw from the action of laccase [12,14]. Another interesting outcome from the fed-batch SSCF supplemented with laccase at 33 hours of fermentation was that no significant variations were found when comparing cell viability between untreated and laccase-treated slurries (data not shown), suggesting that the damage that resulted after exposure of cells to sublethal conditions could not be repaired immediately by reducing the concentration of degradation compounds. In addition, soluble phenols at concentrations of 1.2 g/L and 1.7 g/L were found after 48 hours and 72 hours, respectively. These phenolic values are higher than with the previous strategy (laccase addition at 21 hours) and could increase the synergistic effects of different inhibitors, hindering the fermenting microorganism from adapting to the culture medium.

## Conclusions

The conversion of all fermentable sugars at high substrate loadings can contribute to making lignocellulosic bioethanol production economically viable. In the present work, laccase treatment was employed to overcome the effect of inhibitory compounds from steam-exploded wheat straw and to allow growth of the xylose-fermenting *S. cerevisiae* KE6-12. When wheat straw slurries were treated with laccase before fermentation, the phenolic content was considerably reduced and the slurries could be fermented at substrate consistencies of up to 12% DM (w/v). On the other hand, when using a fed-batch strategy the concentration of inhibitory compounds remains low at the start of the fermentation, allowing *in situ* adaptation of the fermenting microorganism to inhibitors and permitting the fermentation of slurries at 12% DM, even without laccase treatment. Moreover, the hydrolysis process is improved during fed-batch mode, resulting in higher saccharification yields and augmenting the concentrations of fermentable sugars. These benefits can be boosted by laccase supplementation, increasing the consistency of the slurry to 16% DM (w/v), and thereby increasing the ethanol production to 32.3 g/L and the overall yield up to 51%. However, it is very important to optimize the timing of laccase treatment as this will determine the success of the process.

## Methods

### Raw material and steam-explosion pretreatment

Wheat straw to be used as the raw material was supplied by Ecocarburantes de Castilla y León (Salamanca, Spain) and had the following composition (% dry weight): cellulose, 40.5; hemicellulose, 26.1; lignin, 18.1; and others, 15.3 [26].

The material was milled using a laboratory hammer mill in order to obtain small chips of 2 to 10 mm, and these were stored at room temperature until used. Milled biomass was pretreated in a steam-explosion pilot plant carrying a 2 L reactor vessel. The temperature was fixed at 210°C and the residence time was fixed at 2.5 minutes. To obtain saturated steam at 210°C, the pressure inside the vessel was maintained at 19 to 20 bars.

After pretreatment, one portion of collected slurry was vacuum-filtered to recover the liquid fraction and solids were thoroughly washed to obtain the WIS fraction.

### Enzymes

*P. cinnabarinus* laccase (Beldem, Andenne, Belgium) was used for detoxification. The activity (60 IU/mL) was measured by oxidation of 5 mM 2,2’-azino-bis(3-ethylbenzothiazoline-6-sulfonic acid) (ABTS) to its radical cation (ϵ_436_ = 29300 M^-1^ cm^-1^) in 0.1 M sodium acetate (pH 5) at 24°C.

For saccharification, an enzyme mixture of Cellic CTec2 and Cellic HTec2 (Novozymes, Bagsværd, Denmark) was used. Cellic CTec2 is a cellulase preparation that shows high β-glucosidase activity, while Cellic HTec2 is a hemicellulase preparation with mainly endoxylanase activity. Overall cellulase activity was determined using filter paper (Whatman No. 1 filter paper strips; Whatman, Maidstone, UK) and β-glucosidase activity was measured using cellobiose as substrate on Cellic CTec2 cocktail (100 FPU/mL cellulase and 3,950 IU/mL β-glucosidase activity) [36]. Furthermore, xylanase activity was determined using birchwood xylan on Cellic HTec2 cocktail (1,300 IU/mL) [37].

One unit of enzyme activity was defined as the amount of enzyme that transforms 1 μmol of substrate per minute.

### Microorganisms and media

*S. cerevisiae* KE6-12 was used as the fermenting microorganism. This recombinant *S. cerevisiae* strain encoding xylose genes (*XR* and *XDH*) from *P. stipitis* and overexpressing the endogenous xylulokinase has been evolved to grow on lignocellulosic hydrolysates (Albers *et al*., unpublished results) [15]. Pre-inocula were grown for 24 hours at 30°C in 250 mL Erlenmeyer flasks shaken at 150 rpm containing 50 mL of Delft medium as follows: 20 g/L glucose, 20 g/L xylose, 7.5 g/L (NH_4_)_2_SO_4_, 3.5 g/L KH_2_PO_4_, 0.75 g/L MgSO_4_ · 7H_2_O, 2 mL/L trace metal solution, and 1 mL/L vitamin solution. The cells were harvested by centrifugation at 5,000 rpm for 5 minutes at room temperature. The supernatant was discarded and the pellet was washed once with sterile water. The cell pellet was then weighed and diluted with sterile water to obtain the desired inoculum size.

### Liquid fraction fermentation experiments

Pretreated slurry (26% DM w/v) was vacuum-filtered in order to obtain the liquid fraction. The recovered liquid fraction, that non-diluted is equivalent to 26% DM w/v, was further diluted to an equivalent DM concentration of 14% and 12% (w/v) with 0.05 M citrate buffer, pH 5.5. In addition, diluted liquid fractions were supplemented with diammonium phosphate (DAP; 5 g/L). Prior to fermentation, oligomers were enzymatically hydrolyzed at 50°C and 150 rpm for 24 hours to obtain monomeric sugars with an enzyme loading of 0.5 FPU/mL Cellic CTec2 and 2 IU/mL Cellic HTec2. These enzyme doses were selected according to previous optimization studies [38]. The temperature was then reduced to 35°C, and liquid fractions equivalent to 14% DM (w/v) were inoculated with 1, 3, or 5 g/L dry weight cell mass, while liquid diluted to 12% DM (w/v) was only fermented with an inoculum size of 5 g/L. Fermentation tests were performed in triplicate for 120 hours in 100 mL Erlenmeyer flasks shaken at 150 rpm, using rubber caps with a needle to allow outflow of CO_2_.

ANOVA was performed to identify differences in final yields between untreated and laccase-treated liquid fractions. ANOVA proves statistically whether the means of several groups are all different. The confidence level to identify statistically significant differences was 95.0%.

### Simultaneous saccharification and co-fermentation (SSCF) processes

SSCF experiments were run in two different operational modes: batch and fed-batch. For batch SSCF, pretreated slurry was diluted to 12% and 16% DM (w/v) consistencies with 0.05 M citrate buffer, pH 5.5. Fed-batch cultures were performed at an initial substrate loading of 6% DM (w/v) and two pulses of substrate were added at 25 hours and 50 hours, reaching 12% and 16% DM (w/v), respectively, after each addition.

For saccharification, Cellic Ctec2 at 15 FPU/g DM and Cellic HTec2 at 60 IU/g DM were added according to previous optimization studies [38]. As nutrient, DAP at 5 g/L was added. For batch SSCF, 1 g/L *S. cerevisiae* KE6-12 was used for inoculation whereas 3 g/L was used for fed-batch SSCF (taking into account the initial substrate loading, 6% DM (w/v)). In the case of fed-batch SSCF, hydrolytic enzymes and DAP were added together with the substrate in order to keep the concentration of DAP at 5 g/L and enzyme doses at 15 FPU/g DM substrate of Cellic CTec2 and 60 IU/g DM substrate of Cellic HTec2. Moreover, after each addition of substrate, the pH was adjusted to 5.5 with 10 M NaOH.

All the experiments were run in triplicate at 35°C and 180 rpm for 144 hours (batch SSCF) or 168 hours (fed-batch SSCF). In the same way as in the fermentation tests, the SSCF processes were carried out in 100 mL shake flasks, using rubber caps with a needle to allow CO_2_ outflow.

### Laccase treatment

Laccase was used to detoxify the liquid fraction before fermentation tests. For these experiments, *P. cinnabarinus* laccase at 1 IU/mL was added to diluted liquid fraction corresponding to 12% and 14% DM (w/v) after 21 hours of pre-hydrolysis (3 hours before inoculation).

In addition, laccase treatment was also carried out in SSCF experiments either in batch mode or fed-batch mode. When performing batch SSCF, 10 IU/g substrate for laccase enzyme was added to the slurry at 12% or 16% DM (w/v) and incubated for 12 hours at 50°C and 180 rpm before addition of hydrolytic enzymes and yeast. In the case of fed-batch assays, two different laccase addition strategies were studied. *P. cinnabarinus* laccase at 10 IU/g substrate (taking into account the final substrate loading) was added 21 hours after yeast inoculation to a substrate consistency of 6% DM (w/v) or 33 hours after inoculation, when the substrate concentration had reached 12% DM (w/v).

### Analytical methods

Raw material and WIS fraction were analyzed using the standard National Renewable Energy Laboratory (NREL) methods for determination of structural carbohydrates and lignin in biomass [39]. Dry weight of slurry and WIS was determined by drying the samples at 105°C for 24 hours.

Total phenolic content of the supernatants was determined according to a slightly modified version of the Folin–Ciocalteu method [20].

Extracellular metabolites, sugars, 5-HMF, and furfural were analyzed by HPLC using an Aminex HPX-87H column with a 30 × 4.6 mm Micro-Guard Cation-H column (Bio-Rad, Hercules, CA, USA) maintained at 45°C. The eluent was 5 mM H_2_SO_4_ at a flow rate of 0.6 mL/min. Formic acid and acetic acid were determined under the same conditions, while maintaining the column at 65°C.

Samples were taken at different times in the fermentation or SSCF process and centrifuged at 14,000 rpm for 3 minutes. Supernatant was filtered through 0.2 μm nylon filters and stored at -20°C until analysis.

Cell viability was determined as CFU/mL by cell counting on agar plates (20 g/L glucose, 20 g/L agar, 5 g/L yeast extract, 2 g/L NH_4_Cl, 1 g/L KH_2_PO_4_, and 0.3 g/L MgSO_4_ · 7H_2_O). The plates were incubated at 30°C for 48 hours before counting of colonies.

## Abbreviations

5-HMF: 5-hydroxymethylfurfural; ABTS: 2,2’-azino-bis(3-ethylbenzothiazoline-6-sulfonic acid); ANOVA: Analysis of variance; CFU: Colony-forming unit; DAP: Diammonium phosphate; DM: Dry matter; FPU: Filter paper unit; HPLC: High performance liquid chromatography; IU: International unit; NREL: National Renewable Energy Laboratory; SSCF: Simultaneous saccharification and co-fermentation; WIS: Water-insoluble solids; XDH: Xylitol dehydrogenase; XR: Xylose reductase.

## Competing interests

LO does consultancy work for Taurus Energy AB, Lund, Sweden.

## Authors’ contributions

ADM, ETP, DI, MB, and LO participated in the design of the study. ADM and ETP performed the experimental work and wrote the manuscript. LO, DI, and MB conceived the study and commented on the manuscript. All the authors read and approved the final manuscript.
